# The Effects of Macrophage Phenotype on Osteogenic Differentiation of MSCs in the Presence of Polyethylene Particles

**DOI:** 10.3390/biomedicines9050499

**Published:** 2021-05-01

**Authors:** Qi Gao, Claire Rhee, Masahiro Maruyama, Zhong Li, Huaishuang Shen, Ning Zhang, Takeshi Utsunomiya, Elijah Ejun Huang, Zhenyu Yao, Bruce A. Bunnell, Hang Lin, Rocky S. Tuan, Stuart B. Goodman

**Affiliations:** 1 Orthopaedic Research Laboratories, Department of Orthopaedic Surgery, Stanford University School of Medicine, Stanford, CA 94304, USA; qigao7@stanford.edu (Q.G.); cr21@stanford.edu (C.R.); masa1460@stanford.edu (M.M.); hss2018@stanford.edu (H.S.); ningzzz@stanford.edu (N.Z.); takeshiu@stanford.edu (T.U.); eehuang@stanford.edu (E.E.H.); zhenyuy@stanford.edu (Z.Y.); 2 Center for Cellular and Molecular Engineering, Department of Orthopaedic Surgery, University of Pittsburgh School of Medicine, Pittsburgh, PA 15219, USA; alanzhongli@pitt.edu (Z.L.); hal46@pitt.edu (H.L.); tuanr@cuhk.edu.hk (R.S.T.); 3 Department of Microbiology, Immunology and Genetics, University of North Texas Health Science Center, Fort Worth, TX 76107, USA; Bruce.Bunnell@unthsc.edu; 4 The Chinese University of Hong Kong, Hong Kong 999077, China

**Keywords:** MSCs, polyethylene particles, macrophage, osteogenic differentiation

## Abstract

Wear debris generated from the bearing surfaces of joint arthroplasties leads to acute and chronic inflammation, which is strongly associated with implant failure. Macrophages derived from monocytes recruited to the local tissues have a significant impact on bone healing and regeneration. Macrophages can adopt various functional phenotypes. While M1 macrophages are pro-inflammatory, M2 macrophages express factors important for tissue repair. Here, we established a 3D co-culture system to investigate how the immune system influences the osteogenic differentiation of mesenchymal stem cells (MSCs) in the presence of micron-sized particles. This system allowed for the simulation of an inflammatory reaction via the addition of Lipopolysaccharide-contaminated polyethylene particles (cPE) and the characterization of bone formation using micro-CT and gene and protein expression. Co-cultures of MSCs with M2 macrophages in the presence of cPE in a 3D environment resulted in the increased expression of osteogenic markers, suggesting facilitation of bone formation. In this model, the upregulation of M2 macrophage expression of immune-associated genes and cytokines contributes to enhanced bone formation by MSCs. This study elucidates how the immune system modulates bone healing in response to an inflammatory stimulus using a unique 3D culture system.

## 1. Introduction

Total knee and hip arthroplasties are among the most commonly performed surgical procedures in the United States; however, the long-term outcomes of these procedures can be limited by the durability of the implant [1]. One of the complications of these arthroplasties is the accumulation of wear debris from the bearing surfaces, which can lead to implant failure [2,3]. These wear particles are immunologically active and indigestible, thus initiating periprosthetic osteolysis and implant loosening that may necessitate revision surgery [4]. The inflammatory reaction to wear debris plays a crucial role in compromising long-term implant survival, and results from activated macrophages [5,6]. Macrophages have emerged as key components of bone biology and have attracted recent attention in orthopedic research. Dysregulation of the critical interactions and crosstalk between macrophages and mesenchymal stem cells (MSCs) has been demonstrated to cause various pathologies, including fracture non-unions, foreign body reactions, and wear particle disease. In general, uncommitted (M0) macrophages can differentiate into two main phenotypes: M1 or M2 macrophages. M1 macrophages are the initial responders to injury and adverse stimuli and secrete cytokines such as tumor necrosis factor-α (TNFα), interleukin (IL)-1, IL6, and others. M2 macrophages are generally involved in tissue healing, regeneration, and remodeling, mediated through the expression of anti-inflammatory molecules [7]. This differentiation into specific phenotypes depends on local microenvironmental cues, including physical (mechanical), chemical, material, and other signals [8]. Both macrophages and MSCs are also significant sources of cytokines, chemokines, and other substances, thus creating complex signaling pathways and interactions that are critical for maintaining normal tissue homeostasis [9]. To develop and assess candidate strategies for the treatment of wear particle-associated damage, we applied a 3D culture model to investigate the complex interactions of primary human macrophages and MSCs when exposed to polyethylene particles.

## 2. Experimental Section

### 2.1. Cell Culture and Bioreactor Setup

Macrophages were isolated from the buffy coat of whole blood, obtained from deidentified male donors 20 to 40 years of age, using a negative selection protocol with the EasySep™ Human Monocyte Isolation Kit (STEMCELL Technologies, Vancouver, BC, Canada.) [10]. The monocytes were cultured and differentiated in a macrophage-stimulating medium (RPMI (Thermo Fisher Scientific, Waltham, MA, USA) supplemented with 10% FBS (Thermo Fisher Scientific, Waltham, MA, USA), 1% antibiotic-antimycotic (Thermo Fisher Scientific, Waltham, MA, USA), and 100 ng/mL M-CSF (R&D Systems, Minneapolis, MN, USA)) for 5 days to obtain naïve macrophages (M0). The undifferentiated M0 macrophages were polarized to an M1 phenotype by adding 20 ng/mL IFNγ (VWR, Radnor, PA, USA) and 10 ng/mL LPS (Sigma-Aldrich, St. Louis, MO, USA). Then, 20 ng/mL IL-4 (VWR, Radnor, PA, USA) was used to polarize M0 macrophages to an M2 phenotype.

Macrophages were co-cultured with MSCs in a 2:1 ratio in the 3D hydrogel scaffold with or without PE particles coated with 10 ng/mL LPS. MSCs were suspended in hydrogel at a concentration of 8 × 10^6^ cells/mL, and the suspension was photopolymerized using non-ultraviolet light (395 nm). The constructs were cultured in this customized 3D bioreactor with an osteogenic differentiation medium (DMEM (Thermo Fisher Scientific, Waltham, MA, USA), 10% FBS, 1% anti-anti solution, 50 µM L-ascorbic acid (Fujifilm Wako Chemicals USA Corporation, Richmond, VA, USA), 100 nM dexamethasone (Sigma-Aldrich, St. Louis, MO, USA) (days 1–14), 10 mM β-glycerophosphate (Sigma-Aldrich, St. Louis, MO, USA), 100 mM Vitamin D_3_ (Sigma-Aldrich, St. Louis, MO, USA), and 100 ng/mL BMP-7 (Peprotech, Rocky Hill, NJ, USA)). After 4 weeks of incubation, constructs were harvested by sampling with 3 mm skin punch instruments for analysis. Four scaffolds were loaded into one bioreactor. One bioreactor represented one experimental condition. The experiments were replicated (*n* = 2).

### 2.2. qPCR Analysis

RNA was extracted by TRIzol reagent and reverse transcribed into cDNA by the iScript reverse transcription supermix for RT-qPCR (Bio-Rad, Hercules, CA, USA). TaqMan Gene Expression Primers (Thermo Fisher Scientific, Waltham, MA, USA) were used for quantifying the expression of *TNFα* (Hs00174128_m1), *IL-1β* (Hs01555410_m1), *IL6* (Hs00174131_m1), *CCL13* (Hs00234646_m1), *CCL18* (Hs00268113_m1), *RUNX2* (Hs01047973_m1), *osteopontin* (Hs00234160_m1), *osteocalcin* (Hs01587814_g1), and *GAPDH* (Hs02786624_g1). Student’s *t*-tests were used to determine the statistical significance of the qPCR fold changes.

### 2.3. Enzyme-Linked Immunosorbent Assay (ELISA)

Cytokine secretion was analyzed by ELISA according to the manufacturer’s protocols. An ELISA kit of TNFα was obtained from Invitrogen, and the rest were purchased from R&D Systems. Briefly, the captured antibody was coated onto a plate, and then the standards and samples were added. The unbound molecules were washed and removed by a washing buffer. Next, a detection antibody was employed to immobilize our targets. After the addition of the HRP substrate and TMB, absorbance at 450 nm was determined. The Student’s t-test was used to compare the statistic differences between the two groups. Comparison of multiple groups was performed by a two-way ANOVA followed by Dunnett’s test.

### 2.4. Staining and Imaging

The constructs were fixed with paraformaldehyde (PFA) 4% overnight at 4 °C and imaged using a SkyScan 1276 µCT machine (Bruker Scientific Instruments, Billerica, MA, USA). Subsequently, the scaffolds were placed in 15% sucrose (Thermo Fisher Scientific, Waltham, MA, USA), and then 30% sucrose at 4 °C overnight. After being embedded in the OCT compound (Thermo Fisher Scientific, Waltham, MA, USA), the scaffolds were mounted onto a microtome. The scaffolds were sectioned into 10 µm thick slices and stained with hematoxylin (Vector Laboratories, Burlingame, CA) and eosin-Y solution (Sigma-Aldrich, St. Louis, MO, USA). Separate sections were also stained with 40 mM Alizarin Red (Thermo Fisher Scientific, Waltham, MA, USA) and alkaline phosphatase (1-Step™ NBT/BCIP Substrate Solution) (Abcam, Cambridge, UK).

## 3. Results and Discussion

**Figure 1:**
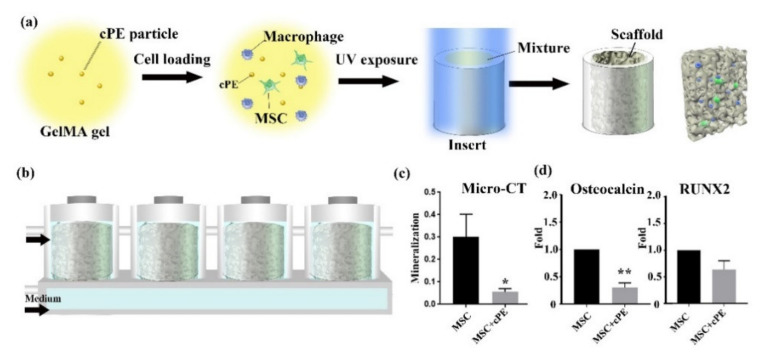
MSCs in 3D culture. cPE decreased bone formation by MSCs. (**a**) Schematic of the bioreactor-based 3D culture system. Cross-section schematic demonstrates cells are embedded in scaffolds. (**b**) Illustration of the custom-designed 3D bioreactor. The medium flows from both top and bottom inlets. Cells are encapsulated in the GelMA scaffold and cultured in the bioreactor. In one bioreactor, 4 scaffolds are connected in series. (**c**) Micro-CT analysis showed a reduction of mineralization in the MSC+cPE group compared to the control MSC group. (**d**) qPCR revealed reduced gene expression of *OCN* and *RUNX2* upon exposure to cPE. (* *p* < 0.05, ** *p* < 0.01).

A 3D cell culture simulates a more authentic in vivo physiologic environment than 2D cell culture conditions. In this study, we cultured cell-laden photo-crosslinked methacrylated gelatin (GelMA) [11,12] in a 3D microphysiological system. These 3D constructs were then cultured in a custom-designed, dual-flow microphysiological system. Figure 1a shows the loading process of the scaffold-based 3D cultures [13]. The medium was perfused into the scaffold chamber from both the upper and lower inlets, as shown in Figure 1b. The wear of conventional metal-on-polyethylene implants generates primarily micrometer-sized polyethylene particles (PE) [14,15]. To model the biological events, such as periprosthetic osteolysis, associated with these particles, constructs with or without PE particles (4.62 ± 3.76 µm [15]) coated with lipopolysaccharides (LPS) (contaminated PE particles, cPE) were cultured for 4 weeks. The mineralization of the tissue was determined by micro-CT scanning. As predicted, we found that the addition of 0.125% cPE significantly decreased bone formation by MSCs, as represented by the mineralization of the tissue shown in Figure 1c. Osteocalcin (OCN) and the transcription factor Runt-related transcription factor 2 (RUNX2) are associated with the osteogenic differentiation of MSCs [16]. The changes in mRNA expression of osteogenic markers in response to cPE are evident by qPCR analysis, as shown in Figure 1d. The qPCR analysis demonstrated significantly decreased expression of *OCN* and reduced expression of *RUNX2* in MSC cultures after the addition of cPE.

**Figure 2:**
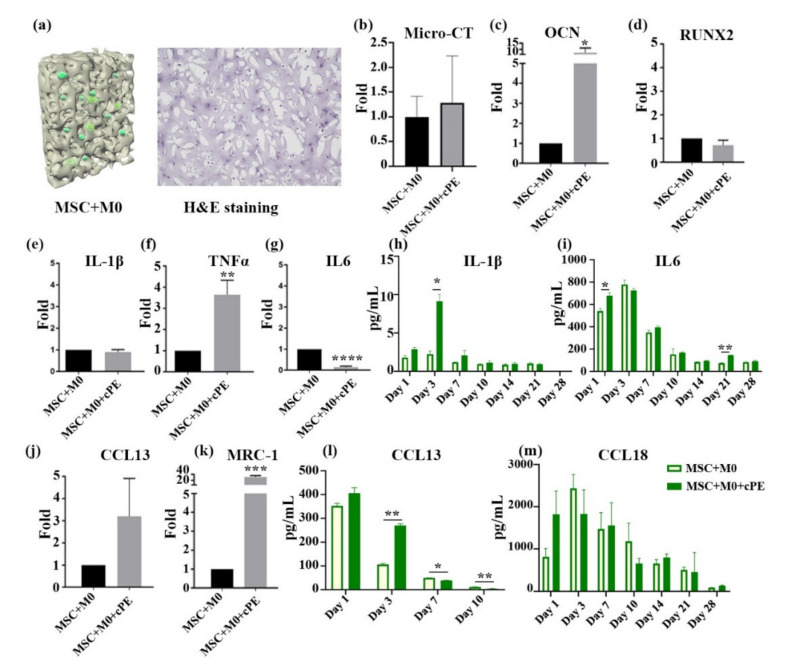
Effects of M0 macrophages on MSC osteogenesis in cPE environment. M0 macrophages polarized to M1 and M2 macrophages and enhanced the osteogenesis of MSCs in the cPE treatment group. (**a**) Cross-sectional illustration of the 3D co-culture. MSCs were co-cultured with M0 macrophages with or without cPE. H&E staining shows cells evenly distributed throughout the scaffold. (**b**) Micro-CT analysis of scaffolds. The amount of mineralization did not differ significantly in the cPE group. (**c**,**d**) qPCR data of osteogenic gene expression. (**e**–**g**) qPCR result of inflammation related genes. (**h**,**i**) Levels of secreted pro-inflammatory cytokines. IL-1β and IL6 were detected. (**j**,**k**) mRNA expressions of *CCL13* and *MRC* were determined by qPCR. (**l**,**m**) Cytokine profile of CCL13 and CCL18 in the medium. (* *p* < 0.05, ** *p* < 0.01, *** *p* < 0.001).

The immune system plays a central role in homeostasis and bone regeneration, mainly through crosstalk between macrophages and MSCs [17]. Studies on the underlying mechanisms of wear debris-mediated osteolysis can provide important insights into bone regeneration in inflammatory conditions and the potential for therapeutic interventions. By incorporating primary macrophages and cPE with primary MSCs, our 3D culture system aims to recapitulate the biological events relevant to the wear of joint replacements. Primary cells were utilized to mimic the in vivo inflammatory response and clinical conditions. As immune cells are known to have increased donor-to-donor variability that may negatively impact the reproducibility of results, primary macrophages of four different donors were used in each experiment to minimize the limitations of individual variations [18,19]. The M0 macrophages were co-cultured with MSCs in a 2:1 ratio in the 3D hydrogel scaffold with or without cPE. Figure 2a illustrates the cross-sectional image with MSCs and macrophages evenly distributed throughout the scaffold. Micro-CT scans were conducted after 4 weeks (Figure 2b) and no significant differences were observed. The qPCR analysis of RNA extracted from the constructs demonstrated the expression of osteogenic markers, as shown in Figure 2c,d. The addition of cPE did not alter the gene expression of *RUNX2* in M0-MSC co-cultures. However, the MSC and M0 macrophage co-cultures showed significantly increased expression of *OCN* after the addition of cPE. This suggests that the presence of M0 macrophages enhanced the osteogenesis of MSCs in the cPE treatment group. We also evaluated the expression of M1 makers, *IL-1β*, *IL6*, and *TNFα*, and M2 markers, *CCL13* and *MRC-1*, via qPCR. Results are shown in Figure 2e–g,j–k. We observed a significantly increased expression of the M2 marker *MRC-1*, and the M1 marker *TNFα*, after the addition of cPE. In contrast, cPE suppressed *IL6*, but did not affect *IL-1β* or *CCL13* production. The presence of cPE not only initiated changes in gene expression but also in cytokine secretion. Particle-activated macrophages produced increased amounts of specific cytokines. The secretion of IL6, IL-1β, CCL13, and CCL18 was analyzed by ELISA. Notably, the co-culture of M0 macrophages with MSCs resulted in increased production of IL-1β, IL6, and CCL18 in the first 3 days. These findings initially increased and then decreased over time. The modestly increased induction of IL6, IL-1β, and CCL18 indicated that M0 macrophages could be polarized towards specific macrophage phenotypes to regulate bone formation. Overall, these results suggested that M0 macrophages polarized to M1 and M2 macrophages.

As a part of the innate immune response, macrophages phagocytose cPE and induce an inflammatory response [20]. A comprehensive investigation of the inflammatory processes associated with particle-associated inflammation is crucial for understanding the mechanistic drivers. M0 macrophages will polarize and assume different functional phenotypes when exposed to specific pro- or anti-inflammatory stimuli and other biological cues [21]. Therefore, macrophages can be classified based on their function and activation. The role of M1 macrophages is to secrete pro-inflammatory cytokines and chemokines and participate in the acute innate immune surveillance and response monitoring system. The primary pro-inflammatory cytokines produced include IL6, IL-1β, and TNFα. M2 macrophages secrete IL-10, CCL13, CCL18, and other anti-inflammatory cytokines, which have the function of reducing inflammation and contributing to wound healing and tissue repair [22]. The levels of both pro- and anti-inflammatory cytokines were enhanced in the M0 co-culture system. This suggests that M0 macrophages were polarized to both the M1 and M2 phenotypes and reflect ongoing MSC–macrophage crosstalk. Understanding these complex interactions in a mechanistic way is likely to provide insights into modulating the osteogenic differentiation of MSCs to enhance bone formation and tissue repair in the presence of adverse stimuli, such as cPE [17].

**Figure 3:**
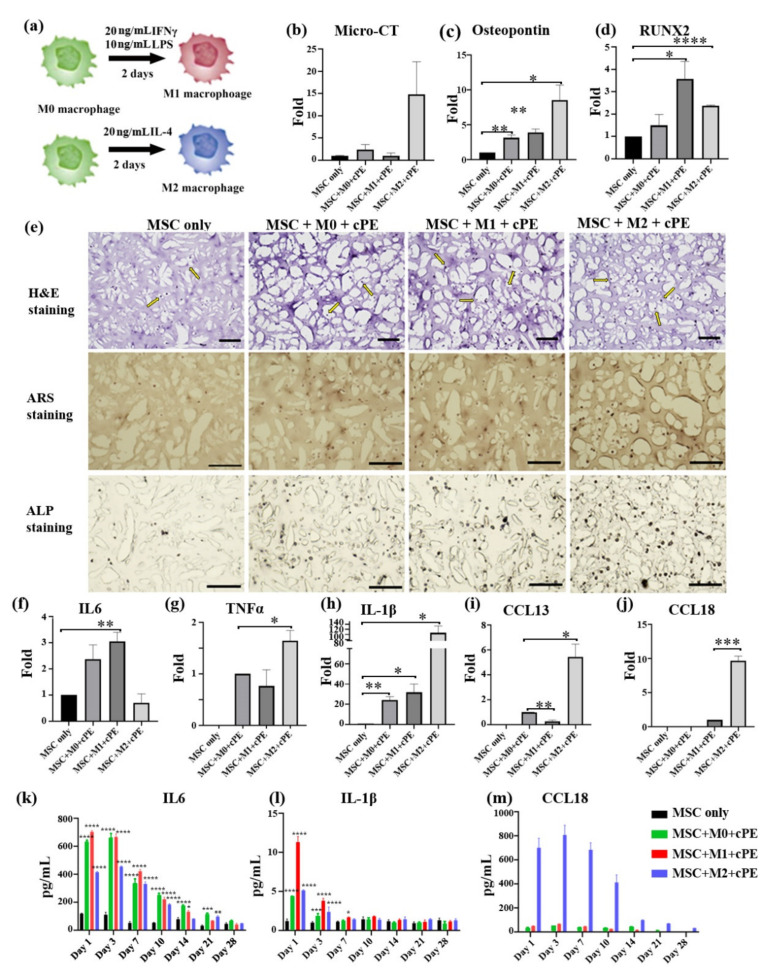
Effects of macrophage polarization on MSC osteogenesis in a cPE environment. M2 macrophages protect the particle-associated effects. (**a**) Schematic of the polarization process. M0, M1, and M2 macrophages were co-cultured with MSCs and cPE. (**b**) Mineralization of the tissue was measured by micro-CT. (**c**,**d**) qPCR data of osteogenic gene expression. (**e**) H&E, ARS, and ALP staining after 4 weeks of osteogenic differentiation (scale bar 100 µm). Yellow arrows indicate cells. (**f**–**j**) qPCR was used to evaluate the expression of *IL6*, *TNFα*, *IL-1β*, *CCL13*, and *CCL18*. (**k**–**m**) Secreted cytokines were detected by ELISA. The MSC-only group was selected as the control group. (* *p* < 0.05, ** *p* < 0.01, *** *p* < 0.001, **** *p* < 0.0001).

Prior research reported that the phenotypes and functions of macrophages are determined in part by local microenvironmental signals and cues. Interferon-gamma (IFN-γ) and LPS are widely used to differentiate macrophages into the M1 pro-inflammatory phenotype. Conversely, IL-4 converts macrophages into the M2 anti-inflammatory phenotype [23,24]. In our experiments, functional macrophages were co-cultured with MSCs and cPE to mimic the clinical scenario. To further evaluate the relationship between macrophage polarization and MSC differentiation and osteogenesis, macrophages were polarized for 2 days before the co-culture experiments, as shown in Figure 3a. M1 or M2 macrophages were mixed with MSCs and loaded in the 3D hydrogel scaffold, respectively. MSC monocultures in the 3D scaffold served as a negative control. The cultures were collected after 4 weeks. As shown in Figure 3b, micro-CT analysis indicated that all macrophage phenotypes enhanced MSC osteogenic differentiation when exposed to cPE; M2 macrophages produced the most significant effects. Osteopontin (OPN), a major glycoprotein produced during bone formation, is secreted by MSCs and macrophages in our co-culture system. mRNA was harvested from the constructs, and it was observed that macrophages promoted the osteogenic differentiation of MSCs when exposed to cPE, as evidenced by the increased expression of *OPN* and *RUNX2* (Figure 3c,d). The cultured constructs were embedded in an optimum cutting temperature (OCT) compound and cryosectioned for assessment of the phenotypic changes resulting from the elevated expression of osteogenic genes. Hematoxylin and eosin-Y (H&E) staining showed that macrophages interacted with MSCs without direct cell-to-cell contact; thus, differentiation appeared to be mainly mediated by soluble factors. Alizarin Red S (ARS) staining was next used to detect the deposition of calcified matrix as an indicator of osteogenic differentiation. In addition, alkaline phosphatase (ALP) was also assessed as another marker of osteogenic differentiation. As shown in Figure 3e, both ALP activity and the deposition of calcified matrix were detected in all the co-culture treatments. However, MSCs from different groups showed different capacities for osteogenic differentiation. Among them, M2 macrophage co-cultures displayed high ALP activity and increased matrix mineralization, suggesting enhanced osteogenic activity. Our results showed that the activation of macrophages toward an M2 phenotype provides a protective effect on the particle-associated effects.

The cultured constructs were subsequently analyzed using qPCR for gene expression of the indicated M1 or M2 markers to assess the underlying mechanisms of macrophage activation in the cPE environment (Figure 3f–j). The expression of cytokines, including *TNFα*, *IL-1β*, *IL6*, *CCL13*, and *CCL18*, was quantified after 4 weeks, with *GAPDH* as the reference gene. Inflammatory factors (*TNFα* and *IL-1β*) and anti-inflammatory factors (*CCL13* and *CCL18*) were markedly upregulated when the M2 macrophage–MSC co-cultures were exposed to cPE. By contrast, IL6 mRNA expression decreased dramatically. Macrophages will be polarized and exhibit distinct functional phenotypes in vivo [25]. Secretion profiles of the macrophages reflect their specific polarization status [26]. Thus, the cytokine expression was further validated with ELISA. ELISA data showed increased secretion of IL-1β, IL6, and CCL18 in all cPE-containing co-cultures over the 4-week time course, compared to MSC groups without macrophages or cPE (Figure 3k–m). A higher expression of IL-1β and IL6 in the M0 and M1 macrophage co-culture system was observed and was markedly influenced by the duration of the culture. In the M2 macrophage co-cultures, macrophages secreted significantly more CCL18 compared with other treatments (CCL18 cannot be detected in the MSC only group). The production of CCL13 was higher in the M2 macrophage co-culture group during the first 10 days (data not shown).

**Figure 4:**
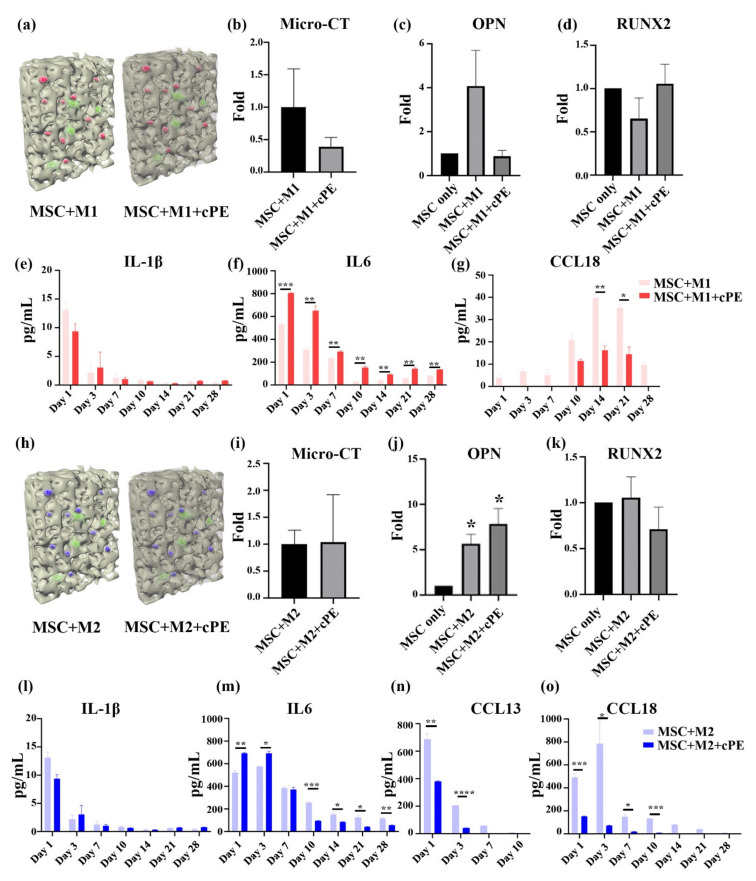
Effects of macrophage polarization on MSC osteogenesis after 4 weeks of co-culture in 3D scaffolds. (**a**–**g**) M1 co-cultures. (**a**) Cross-sectional illustration of the M1 macrophage–MSCs co-culture. (**b**) Mineralization of the tissue quantified by micro-CT. (**c**,**d**) Osteogenic gene expression analyzed by qPCR. (**e**–**g**) Cytokine secretion profiles of IL-1β, IL6, and CCL18 analyzed by ELISA. (**h**–**o**) M2 co-cultures. (**h**) Cross-scheme illustration of the M2 Macrophage–MSCs co-culture. (**i**) Mineralization of the tissue quantified by micro-CT. (**i**–**k**) Osteogenic gene expression analyzed by qPCR. (**l**–**o**) Cytokine secretion profiles of IL-1β, IL6, CCL13, and CCL18 analyzed by ELISA. (* *p* < 0.05, ** *p* < 0.01, *** *p* < 0.001, **** *p* < 0.0001).

Wear particles can alter the macrophage functional phenotype, including cytokine expression. The upregulation or downregulation of cytokines is associated with different macrophage functions and regulates bone regeneration. To explore the cPE-elicited inflammation, MSCs were co-cultured with M1 and M2 macrophages with or without cPE. Micro-CT and qPCR were conducted to compare the potential for osteogenic differentiation, as shown in Figure 4a–d. The trends suggested that the osteogenesis was not changed by the addition of M1 macrophages. In contrast, the results in Figure 4h–k showed that the M2 macrophage increased *OPN* expression significantly, regardless of the presence of cPE. ELISA analysis was conducted to compare cytokine secretion. M1 co-cultures expressed higher levels of IL-1β and IL6 than M1 co-cultures in the presence of cPE in the scaffold. Enhanced IL6 production was detected from M1 macrophages with cPE, indicating that cPE promoted inflammatory activation. The results in Figure 4g demonstrate little change in CCL18 secretion activity between day 1 and day 7, followed by a significant increase. However, M1 macrophages co-cultured with cPE showed a reduced expression of CCL18, suggesting that cPE induced a pro-inflammatory response while suppressing the polarization of M1 macrophages to an M2 phenotype. MSCs co-cultured with M2 macrophages showed higher levels of CCL13 and CCL18. However, there was decreased secretion of CCL13 and CCL18 in cPE-containing scaffolds, whereas IL6 was higher in the cPE condition than in M2 co-cultures. Cytokine production decreased over the culture period (Figure 4l–o). Interestingly, few M2 macrophages changed their polarization status during the culture process. These results suggest a protective effect of M2 macrophages on bone regeneration in an inflammatory microenvironment.

## 4. Conclusions

Macrophages play a critical role in inflammation, bone healing, and tissue regeneration. The addition of an inflammatory stimulus decreased the ability of MSCs to undergo osteogenesis, as evidenced by micro-CT and qPCR data; notably, the combination of all the macrophage phenotypes protected against this adverse effect. Accumulating evidence indicates that wear particles interfere with osteogenic differentiation of MSCs, significantly disturbing bone homeostasis. A 3D co-culture system was established to investigate how the innate immune system influences the osteogenic differentiation of MSCs in an adverse inflammatory environment. The ELISA results showed that particles stimulated an inflammatory response from the macrophages; in co-cultures with MSCs the anti-inflammatory activities were reduced. Interestingly, the macrophage–MSC co-cultures enhanced osteogenic differentiation. Co-cultures with M2 macrophages resulted in the improved deposition of calcified bone matrix, suggesting improved bone maintenance. Thus, the upregulation of immune process-associated genes by M2 macrophages could contribute to enhanced bone formation by MSCs in adverse conditions involving wear particles. By applying a bioreactor to establish a co-culture system of macrophages and MSCs, important insights into the mechanisms of wear particle-induced inflammation were investigated. The modulation of macrophage phenotypes in 3D co-cultures with MSCs represents a potentially useful approach to developing strategies to enhance bone healing and tissue regeneration.

## Data Availability

Not applicable.
